# Use of low dose of rFVIIa (recombinant Factor VII activated) to control late bleeding after percutaneous dilational tracheostomy

**DOI:** 10.1002/ccr3.2061

**Published:** 2019-02-19

**Authors:** Dario Nicosia, Antonino Federico, Ivan Vigna, Pasquale Iozzo, Giovanni Misseri, Andrea Cortegiani

**Affiliations:** ^1^ Department of Surgical, Oncological and Oral Science (Di.Chir.On.S.), Section of Anesthesia, Analgesia, Intensive Care and Emergency, Policlinico Paolo Giaccone University of Palermo Palermo Italy

**Keywords:** activated factor VII, percutaneous tracheostomy, postoperatory bleeding, rFVIIa, Thrombocytopenia

## Abstract

In our case, the use of a low intravenous bolus dose of rFVIIa (recombinant factor VII activated; 15‐20 mcg/kg) was effective and uneventful in controlling late postprocedural PDT bleeding associated with thrombocytopenia that cannot be corrected and after all other treatments failed.

## 1 INTRODUCTION

Bleeding is the most frequent perioperative and postprocedural complication after percutaneous dilational tracheostomy (PDT), especially in patients with comorbidities. We present a case of late post‐PDT bleeding due to persistent thrombocytopenia that could be controlled with a single low dose of recombinant factor VII activated, after other treatments failed.

Patients may need tracheostomy for prolonged mechanical ventilation. Since the beginning of 90s, percutaneous dilational tracheostomy (PDT) has become a common bedside technique for critically ill patients needing a tracheostomy.^1^ Bleeding was the most common complication, with a reported incidence of around 5%.^2^, ^3^ Late bleeding may also occur due to coagulopathies and/or multi‐organ failures disease.^3^, ^4^ The effect of activated factor VII (FVIIa) is based on its effect on tissue factor exposed on the subendothelium. Once the complex FVIIa‐tissue factor complex is formed at the site of endothelial injury, activation of factors IX and X and the formation of thrombin lead to local hemostasis. Then, thrombin triggers platelet activation and consequently factors V and VII.^5^

Recombinant factor VII activated (rFVIIa) is an agent approved in both hemophilia A and B patients, factor VII deficiency, and Glanzmann's thrombasthenia.^6^ It has also been used in several surgical conditions to stop persistent bleeding in patients without inherited coagulopathies.^6^

We report the case of a man with amyotrophic lateral sclerosis (ALS) admitted to our general intensive care unit (ICU) for respiratory failure and presenting a late postprocedural bleeding after 36 hours from PDT due to thrombocytopenia.

## 2 CASES DESCRIPTION

A 80‐year‐old man, suffering from ALS and in chronic domiciliary noninvasive ventilatory therapy, was admitted to the intensive care unit of the University General Hospital (Policlinico Paolo Giaccone) in Palermo, Italy, on July 2018, because of a respiratory failure associated due to Klebsiella pneumoniae pneumonia. His past medical history included hypertension, diabetes, and episodes of pancreatitis.

After have been intubated and mechanically ventilated, he started an empiric and, later on, targeted antibiotic therapy. The patient showed clinical and laboratory signs of resolution of the infection, after 10 days of antibiotic therapy. However, he failed several attempt of weaning from invasive mechanical ventilation. On day 15th after admission, after obtaining informed consent, we performed a PDT according to Griggs's technique under fiberoptic broncoscopy guide. At the time of the procedure, platelet count was 55 × 10^3^ µL and the hemoglobin (Hb) level was 8.9 g/dL. The procedure was uneventful, and there were no peri‐procedural complications in the first 36 hours.

**Figure 1:**
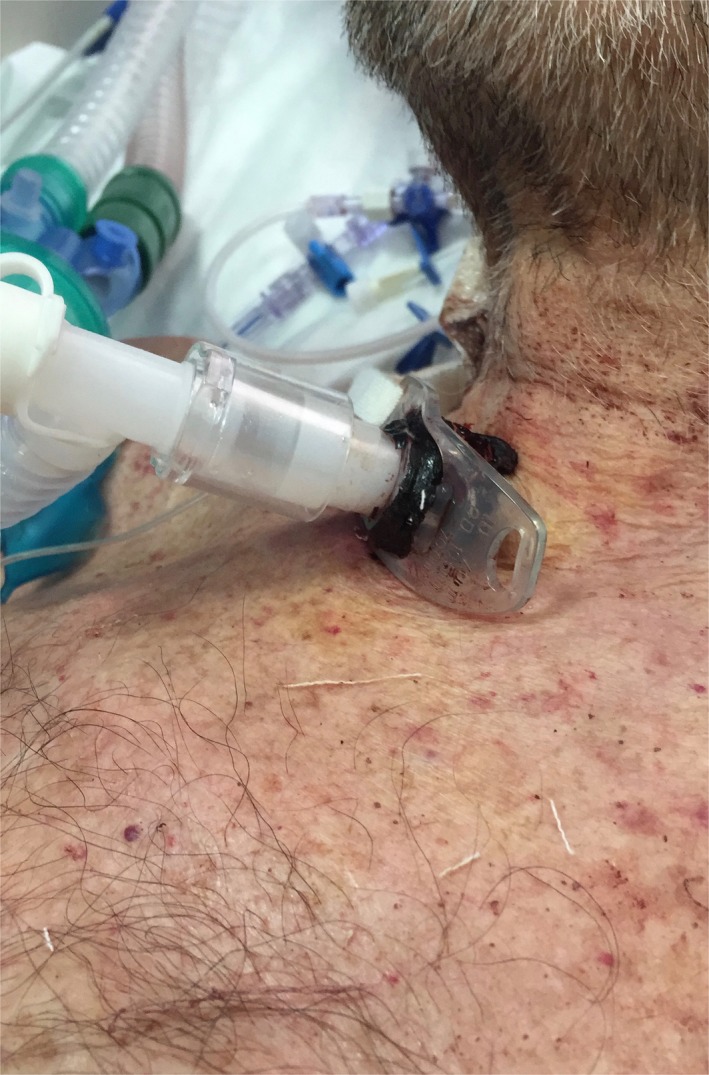
This picture shows peri‐tracheostomy clotted blood without residual bleeding 30 min after the administration of rFVIIa

On day 17th after admission, a continuous low‐grade bleeding started from the tracheostomy. It caused patient's discomfort and required several changes of the surgical dressing and tracheal suctionings. Laboratory tests revealed an Hb level of 7.6 g/dL and a platelet count of 35 × 10^3^ µL. However, other first‐line coagulation tests within the limits, including fibrinogen. Bleeding lasted for about 72 hours, and the patient requires a packed red blood cells (PRBC) transfusion. Administration of methylprednisolone 125 mg once a day was started on hematologist consultancy to increase platelet count. On day 20th, the patient received a transfusion of a pooled platelet unit. In the same day, the patient could not be transfused with additional platelet units, although a platelet count of 20 × 10^3^ µL because of a serious shortage of blood components. Bleeding continued and clinical status and blood tests did not improve significantly despite correction of hypocalcemia and acid‐base balance, normotermia, tranexamic acid administration, and a serum fibrinogen level >200 mg/dL. On day 22 after admission, we started a second‐line bypassing therapy with a single‐bolus administration (15 mcg/kg) of recombinant activated rFVIIa. Interestingly, the peri‐tracheostomy bleeding stopped in about 30 minutes from the administration of rFVIIa without any side effects (Figure 1).

The patient continued cortisonic therapy for other 7 days. On day 30th after admission, after normalization of platelet count (52 × 10^3^ µL) the patient was discharged to a step‐down unit in invasive mechanical ventilation via a portable home care ventilator unit.

## 3 DISCUSSION

In our case, a low intravenous bolus dose of rFVIIa stopped a late, uncontrollable peri‐tracheostomy bleeding, associated with thrombocytopenia.

Since its commercially availability in 1995, recombinant activated factor VII (rFVIIa) has been used a rescue therapy in several cases where conventional therapies failed.^6^ The “on label” use in Europe is intended for patients with hemophilia A and B, for treatment of congenital factor VII deficiency and for Glanzmann's thrombasthenia.^5^ However, the use of rFVIIa has been also extended to those cases in which patients presented thrombocytopenia, platelet function abnormalities and warfarin over‐anticoagulation.^7^ Moreover, it has been used in various conditions such as adult and pediatric cardiovascular surgery, obstetrical hemorrhage, trauma, intracranial hemorrhage, gastrointestinal bleeding, urologic surgery, and transplantation.^6^, ^8^, ^9^ It is not associated with the risk of blood‐transmitted diseases, as a recombinant product is not derived from human or animal plasma.^10^ Thrombosis is one of the most serious complications. However, it has been shown that even high doses as 346 mcg/kg may be well tolerated in hemophilic patients.^10^ The reported incidence of thromboembolic event ranged from 1% to 2% up to 9.8%.^11^ A retrospective study analyzing the use of rFVIIa between 2000 and 2008 showed that in 97% of the time it was used “off label”.^8^

Different systematic reviews, retrospective studies, and case series on the efficacy of low “off label” intravenous bolus dose of rFVIIa (20‐40 mcg/kg) founded that this range of dose was efficacy in controlling or stopping bleeding in major and minor surgical procedures.^6^, ^8^, ^9^, ^12^ However, its “off label” use should be limited only when the bleeding does not respond to conventional medical therapies and/or transfusions^13^ considering the risk of thrombotic events and high cost. It should be noted that although no specific guidelines for management of post‐PDT bleeding are available, the last version of the European Society of Anaesthesiology (ESA) guidelines for management of perioperative bleeding suggested that the use of rFVIIa could be considered for “bleeding which cannot be stopped by conventional, surgical or interventional radiological means and/or when comprehensive coagulation therapy fails”.^13^ In our case, correction of confounding factors (ie, acid‐base unbalance, hypocalcemia, hypothermia), administration of tranexamic acid, and correction of hypofibrinogenemia were performed according to guidelines, in association with a first platelet unit transfusion.^13^, ^14^ However, these measures did not stop bleeding leading us to consider the use of rFVIIa. Since the administration of rFVIIa does not resolve the cause of bleeding per se, it should be considered as a “not specific” form of coagulation support. This agent should not be considered a suitable option for all type of post‐tracheostomy bleeding due to the wide range of potential causes, from erosion of veins (thyroid, brachiocephalic, anterior jugular communicating veins) to arterial fistula.^3^ In our case, the low‐grade continuous stomal bleeding after 36 hours, in association with thrombocytopenia, which is one of the most important risk factor for “chronic bleeding,” limited the differential diagnosis. The lack of further available platelet units for transfusion led us to consider the use of rFVIIa to stop bleeding in our critically ill patient.

The main limitation of this case was that we did not perform viscoelastic coagulation tests (eg, thromboelastography‐TEG, ROTEM).^13^ The viscoelastic techniques could have helped us to understand other causes of bleeding in presence of thrombocytopenia, leading to a more specific coagulation support related to the underlying cause.

## 4 CONCLUSION

In our case, a single‐bolus low dose of rFVIIa was effective and uneventful in controlling late postprocedural PDT bleeding associated with persistent thrombocytopenia, after all other treatments failed.

## CONFLICT OF INTEREST

The authors declare no conflict of interest.

## AUTHOR CONTRIBUTION

DN, AF, IV, PI, GM, AC: treated the patients, helped retrieving the data, and conceived the content of the manuscript. IV, PI: helped retrieving the data and helped writing the manuscript. DN, AF, IV, PI, GM, AC: wrote the manuscript. All authors read and approved the final version of the manuscript.
